# Midwives' experiences working with women and girls surviving violence in Yemen: a qualitative study

**DOI:** 10.3389/fgwh.2025.1450053

**Published:** 2025-03-21

**Authors:** Marwah Al-Zumair, Luz Marina Leegstra, Hussein Zaid, Raisa Ferrer Pizarro, Monia Al-Zumair, Lamya Bawahda, Albrecht Jahn, Lauren Maxwell

**Affiliations:** ^1^ Heidelberg Institute of Global Health, University of Heidelberg Faculty of Medicine, Heidelberg, Germany; ^2^ Department of Communications, Pontificia Universidad Católica del Perú, Lima, Perú; ^3^ School of Medicine and Health Science, Emirates International University, Sana’a, Yemen; ^4^ National Yemeni Midwives Association, Aden, Yemen

**Keywords:** midwives, violence against women, qualitative research, Yemen, child marriage, sexual and reproduction health, reporting violence

## Abstract

**Background:**

Yemeni women and girls have long endured pervasive violence, a situation further exacerbated by the ongoing humanitarian crisis. Violence against women and girls (VAWG) is strongly stigmatized in the Yemeni context. In under-resourced, rural settings like Yemen, where gender inequities prevent women and girls from accessing the formal health system, community midwives may be an important resource for women and girls who experience interpersonal violence. This study explored community midwives' knowledge, training, and applied experience working with women and girls who experience interpersonal violence.

**Methods:**

We conducted 20 in-depth interviews with community midwives in four Yemeni governorates. A female Yemeni physician and qualitative researcher trained in the ethical conduct of VAWG-related research conducted interviews using a semi-structured interview guide. Participants gave verbal consent for participation in the one-time interview. We used thematic analysis to summarise the findings. Interviews were transcribed in Arabic and English, and differences in interpretation were resolved through consensus.

**Results:**

While midwives had limited formal training in supporting women and girls who experience interpersonal violence, they play a critical role in responding to VAWG in Yemen. Community midwives provide psychological support, contraception, violence-related health care, and referrals to more advanced healthcare and protection services, including women-friendly spaces (WFSs) and shelters. Lack of training and treatment guidelines, in addition to a lack of supportive services and VAWG-related stigma, were important barriers for midwives working with VAWG. The stigma associated with sexual violence discouraged women from seeking health care or accessing limited protection services.

**Conclusion:**

Community midwives in Yemen are well-placed to support women and girls who experience violence. Midwives should receive context-appropriate training and support to work with women and girls who experience violence. The lack of available services and the stigma associated with experiencing, reporting and supporting VAWG survivors must be carefully considered before designing any intervention.

## 1 Introduction

Violence against women and girls (VAWG) is a pervasive global public health and human rights issue (1, 2). Sexual violence and intimate partner violence (IPV) are among the most prevalent forms of VAWG (3). VAWG is defined as any act of gender-based violence that causes, or is likely to cause, physical, sexual, or psychological harm or suffering to women and girls (4). This includes acts perpetrated by individuals, including (IPV) and family violence, as well as community-level violence, such as harassment or abuse by colleagues, co-workers, or other community members (5). VAWG encompasses systemic deprivations of women's liberty and rights, such as denial of education, early or forced marriage, and limited access to sexual and reproductive health services (4). VAWG is associated with myriad adverse short- and long-term health outcomes and affects educational and economic achievement and women and girls' sexual and reproductive health (SRH) and rights (6). VAWG-related health complications can be short-term, like injuries, bleeding, and anxiety, or long-term, like depression, vaginal fistula, and sexually transmitted diseases (7–10). Before the 2014 war, 49% of Yemenis lived in multidimensional poverty (11). The war and ensuing cholera epidemics, flooding, drought, and pandemic have devastated Yemen's economy, health system, and public infrastructure. Yemen represents the world's largest humanitarian crisis, with two-thirds of its population (approximately 20.7 million individuals in December 2017) dependent on humanitarian aid (12). In addition to conflict-related violence, women and children are at heightened risk of different forms of family violence, including child early and tourists marriage; child labour, trafficking, family separation; gendered access to limited food, educational opportunities, and medical care; and intimate partner and family physical, sexual, and reproductive violence (7, 8).

The 2013 Yemeni Demographic and Health Survey (DHS), the only DHS conducted in Yemen since 1992 and the only DHS to ask violence-related questions, found that 92% of women and girls ages 15–49 reported that violence was common within their homes (13). A report by the United Nations Office for the Coordination of Humanitarian Affairs (UNOCHA) found a three-fold increase in early child marriage for girls under 18 in Yemen from 2017–2018 (14). Other studies identified an increase in trafficking and other forms of sexual exploitation during the crisis (9). While the 2013 Yemen DHS reported that the median age of marriage was 18 (13), smaller-scale studies have found an increase in child marriage after the initiation of the 2014 war as families that are no longer able to feed their children see child marriage as an acceptable compromise (9). Tourist marriage, a novel form of sexual exploitation where girls are temporarily married to affluent men from the Gulf Arab region, occurs during summer when there is a significant influx of tourist from Gulf countries visiting Yemen. Subsequently, the groom leaves the country and annuals the marriage without communication with the bride, her family, or Yemeni authorities. Tourist marriage has emerged in response to the financial pressures created by the longstanding economic crisis (15).

While limited studies have assessed VAWG following the initiation of the 2015 conflict (16) global research suggests that VAWG increases in severity and prevalence during conflicts (17) and the conflicts will likely have increased the spectrum, severity, and incidence of VAWG in Yemen (7, 9). In alignment with global patterns numerous studies in comparable contexts have highlighted correlations between displacement, financial hardship, early and forced marriage, and limited access to education and health services for women. For instance, findings from South Sudan, Syria, and Palestine have documented a significant increase in the prevalence of early child marriage, driven by protracted conflict and deeply entrenched gender-inequitable cultural norms (18–21). Additionally, a recent systematic review on the prevalence of sexual violence among female refugees and internally displaced persons (IDPs) across 14 countries reported a 21% increase in incidents of sexual violence, underscoring the heightened risk of GBV during humanitarian emergencies (22). The main determinants of VAWG in Yemen and similar settings such as South Sudan and Northern Uganda include poverty, gender inequitable norms, laws, and policies, social fragmentation during crises, economic and familial instability, and the collapse of public institutions. These shared challenges highlight the intersection of conflict, gender-inequitable cultural norms, and systemic inequalities in perpetuating VAWG (18, 23).

### 1.1 Role of community midwives in identifying and addressing VAWG

There are several important movements to unite Yemeni women and girls to address both SRH and VAWG, including the National Yemeni Midwifery Association (NYMA). NYMA was founded in 2004 with over 3000 members from all 22 Yemeni governorates. For most Yemeni women and girls, community midwives are their only source of healthcare. In collaboration with international partners, NYMA has provided midwives with training on critical SRH topics, including family planning, emergency obstetric care, and women's health and empowerment. The association has also supported midwives in establishing private clinics to enhance access to maternal and reproductive healthcare. Additionally, NYMA has led health promotion campaigns aimed at educating women and girls on the health-related complications of early marriage, raising awareness about violence, and advocating for women's rights. Several challenges hinder NYMA's ability to comprehensively address SRH needs in Yemen. These challenges include limited funding opportunities, inadequate opportunities for midwifery training, particularly in rural areas, and insufficient education on VAWG due to societal stigma and public resistance to such training. Despite these restrictions, NYMA remains committed to advocating for greater investment in midwifery education, the implementation of targeted training programs, and enhanced support to ensure equitable access to life-saving maternal and child health (MCH) services across the country (20).

For survivors of sexual violence, midwives can play a significant role in managing injuries, stopping bleeding, and facilitating women's access to emergency contraceptives and post-exposure prophylaxis (PEP). Midwives can provide VAWG survivors with information and support to help them access community resources and connect victims with psychological, legal, police, shelter, and financial services (24, 25). Midwives can also raise awareness about early marriage, contraceptive counselling, and SRH rights during field visits (26).

### 1.2 Resources for Yemeni women and girls who experience violence

Preventing VAWG requires strategic approaches that involve coordinated community efforts, effective resource allocation, and adequate funding (27, 28). Disclosing abuse and obtaining support can reduce posttraumatic stress, self-blame, and revictimisation (29). Psychological support, risk assessment, safety planning, home visits, and referral to supportive care are examples of women-centred VAWG-related interventions (28). Some evidence shows that domestic violence counselling, advocacy, and hotline services can improve self-efficacy and coping skills for women who experience IPV (30). Shelters can offer a safe refuge for women and their children, providing access to social, legal, medical, and economic assistance within a safe environment (30, 31). There is limited evidence on the efficacy of protection services in humanitarian settings, and no studies have explored barriers to accessing interpersonal violence-related services or service efficacy in Yemen (25, 32).

Several initiatives exist to address VAWG in Yemen. As part of UNFPA's response to VAWG, in 2024, 37 safe spaces run by the UN and their partners across 20 governorates provided multi-sectoral GBV services, including vocational training supporting over 22,951 women. Eight shelters in 7 governorates offered immediate safety and life-saving services for GBV. Seven psychological support centres reported nearly 100,000 individuals accessed mental health hotlines, with 75% identified as survivors of violence (33, 34). VAWG services are also carried out in women-friendly spaces (WFSs), supported by UNFPA and their partners to assist and empower vulnerable women and girls. Safe spaces are often integrated into existing community structures such as schools or health. In these spaces, women can receive psychological, legal, and medical support and be trained in livelihood skills (35–38). Reports from UNFPA partners in 2024 highlighted various initiatives in WFSs, including campaigns to address VAWG and economic grants to support women's vocational training in areas such as sewing and incense-making. Additionally, these spaces organised awareness sessions on early marriage and women's rights (39, 40). The Yemeni Women's Union (YWU) also operates multiple shelters for VAWG survivors, offering legal, social, and economic support. Similar to WFSs, these shelters aim to protect survivors and facilitate their reintegration into society empowering them to rebuild their lives with dignity and independence (37, 38, 41).

While limited evidence exists on the overall effectiveness of VAWG-related interventions, UNFPA and other GBV partners shared several success stories where women, with the support of these interventions, were able to escape abusive relationships and rebuild their lives (35, 37, 42). Other VAWG-related services address complications such as traumatic birth injuries, including obstetric fistula, a condition primarily associated with early child marriage, premature childbearing and restricted access to SRH services (43). As such, obstetric fistula is associated with VAWG, principally child marriage and limited access to healthcare, and causes violence by leading to social and economic isolation and other forms of stigma and violence (44). UNFPA provides free surgical treatment and medical care to over 100 women annually suffering from obstetric fistula (45). VAWG services are often integrated with emergency reproductive health programs, delivered by mobile teams and social health workers trained to support survivors or refer them to additional resources, including shelters (41). Despite these resources, support for VAWG survivors remains critically insufficient, largely due to limited individual and communal resources, the stigma associated with seeking help, and widespread societal opposition to VAWG-related interventions (46).

### 1.3 The challenge of understanding and addressing violence

Access to comprehensive VAWG-related interventions remains inadequate and fragmented. Services are predominantly concentrated in urban areas, leaving the 90% of the population in rural regions underserved (47). The diversion of donor funding due to conflict and the COVID-19 pandemic further reduced the scope of VAWG-related activities, including cash assistance programs and shelters (48). In 2021, 12 WFSs were closed, resulting in 350,000 women and girls losing access to essential services (49). Survivors with disabilities or those from minority groups face heightened challenges due to intersecting vulnerabilities, including discrimination and extreme poverty (47). Additionally, stigma, insecurity, and fear of traveling, lack of permission to travel, or no financial resources to travel, to service centers deter many survivors from seeking help (46). A lack of information about available interventions, women and girls' restricted mobility, and societal stigma associated with VAWG-related initiatives further limit access to support. Negative perceptions of VAWG also constrain research, funding, and opportunities to address VAWG in Yemen (46).

### 1.4 Study objectives

VAWG is a highly stigmatized and under-researched topic in Yemen. We conducted a systematic review on VAWG in Yemen and found that very few studies have addressed VAWG post the 2015 conflict or included healthcare providers and no studies with midwives (16) Despite an increase in violence against women (VAW) during wartime (9), the involvement of healthcare providers in VAWG response, along with their barriers and available resources, has not been researched in Yemen. Researchers in Yemen often avoid this topic due to challenges in recruiting participants, securing partnerships, obtaining funding or ethical clearance, and supporting research teams. Consequently, key aspects of VAWG in Yemen, such as its relation to health and reproductive outcomes, existing resources, and the barriers and facilitators faced by healthcare providers, remain largely unexplored. Women face significant challenges when accessing VAWG-related services, the barriers faced by midwives who work with women and girls who experience violence have not been studied. Understanding the broader context of VAWG in Yemen, as well as the challenges and risks faced by both midwives and the women and girls they serve, is of critical importance for preventing, mitigating, and measuring VAWG.

We conducted a cross-sectional, qualitative study to: (1) understand Yemeni midwives' responses and experiences in addressing VAWG, (2) the training they have received, (3) barriers for midwives to report the violence they witness or learn about or for women and girls who experience violence to report their experience, and (4) the available resources external to the family, facilitators, and challenges to accessing VAWG services.

## 2 Methods

We conducted one-time, in-depth interviews (IDIs) with 20 community midwives in four Yemeni governorates. These four governorates were strategically selected based on their accessibility to the research team and their close ties to NYMA. Although additional governorates, such as Al Hudaydah and Hadramout, were initially considered for inclusion, safety concerns and accessibility challenges prevented us from conducting the study in these governorates. The selected governorates encompass the northern, southern, and central regions of Yemen, ensuring a more comprehensive representation of the country's cultural diversity, which may affect the manifestations of VAWG, access to resources, or pathways for addressing violence.

We conducted a systematic review of the types of VAWG in Yemen (16) that we used to inform the development of the IDI guide. An expert in VAWC and SRH-related research and a Yemeni SRH-focused physician and qualitative researcher developed the semi-structured interview guide. We piloted the interview with three Yemeni midwives and the head of NYMA. Following the pilot, we shortened the interview guide and rephrased how we discussed women's shelters. The interview guide included questions and probes to understand the role midwives play in responding to VAWG, barriers they face in supporting survivors or reporting abuse, the training they received to support women and girls who experience violence, resources available to women and girls who experience violence and perceived or known barriers that could prevent them from accessing those services. Fifteen interviews were conducted face-to-face, and five were over the phone due to war-related travel disruptions.

The following questions were included in the interview guide:

1. What types of violence against women have you seen in your community or during work in SRH?
2. What training have you received to work with women who experience violence?
3. If a woman tells you that she is experiencing violence, what do you do?

Probe:

• Report the violence?
  • To whom?
  • With her permission?
  • With permission from your work? Explain, how is the process of reporting incidence of violence in your facility?
• Refer the woman to services
  • What services
  • Where?
  • How open is access to those services?
• Refer the woman to another professional
  • Who?
  • How available is that support?

4. What worries you the most about talking to the women or girls about the violence they experience?
5. What barriers or challenges have you faced while working with women experiencing violence?
6. Have you ever been afraid for your safety when working with a woman or child who experiences violence?
7. What are the services available to women and girls who experience violence?
8. Are there specific helplines or resources, such as psychological support or family counseling, that women who experience violence can access?
9. What do you know about the resources available to empower women?
10. What are some of the barriers to accessing services?
11. How have the support and services that women who experience violence can receive changed after the war?

### 2.1 Participant recruitment and inclusion criteria

Research participants were midwives who currently worked or had worked as part of NYMA. NYMA leadership invited midwives from different governorates representing urban and rural settings to participate in the IDIs. All midwives who were invited agreed to participate.

### 2.2 Data capture and analysis

Interviews were conducted in Yemeni Arabic, lasted between 1 and 1.5 h, and were completed in one session. We recorded in-person IDIs with a digital recorder and remote IDIs by phone. Digital recordings were transcribed verbatim from spoken into written Arabic and subsequently translated into English. Differences of opinion in the translation were resolved through consensus. Identifying information was removed from the written transcripts. De-identified transcripts were uploaded to MAXQDA, Version 2.4 (50), for analysis. Initial interviews were discussed on a rolling basis to identify emerging interpretations and refine the interview guide. Subsequent interviews needed to be completed quickly during a field visit to Yemen, and transcription and analysis happened after the interviews were completed.

We conducted a thematic analysis grounded in Colaizzi's model of descriptive phenomenology (51). During the first phase of the analysis, we developed an initial set of deductive codes and definitions based on (1) the interview guide, (2) literature identified through a systematic review, search strategy detailed in Supplementary Table S1, and (3) our experience as VAWC researchers and MCH care providers. We subsequently developed inductive codes by reviewing interviewer notes, transcripts, and analytic memos. The team read transcripts several times to build familiarity with the data and jointly develop the codebook. MAL, HA and LL met weekly to discuss interview coding and refine the codebook. At least two team members independently reviewed all transcripts, and the four core team members (MAL, HA, LL, LM) reviewed and evaluated a subset of transcripts. The entire analysis team met monthly to group the codes into meaningful themes. We stopped conducting interviews after reaching saturation, defined as not identifying new themes (52). Codes are presented in Supplementary Table S2; themes and related quotations are included below and in Supplementary Table S3. We provide the original Arabic-language version of all English-language translations of quotes included in this manuscript in Supplementary Table S3. Results are reported in keeping with the Standards for Reporting Qualitative Research (53).

#### 2.2.1 Reflexivity

Participants were interviewed by a Yemeni obstetrician and global health researcher trained in qualitative research. The research team consists of researchers from Yemen, Germany, Peru, the US, and Argentina. The corresponding author had lived in Yemen and learned Yemeni Arabic. Most authors have experience in community-based participatory research methods, and several authors are experts in sexual and reproductive health rights (SRHR).

## 3 Ethical considerations

The research protocol and related documentation were approved by the Ethics Review Committee of both the University of Heidelberg and Sana'a University. Interview participants provided verbal informed consent because the COVID-19 pandemic was ongoing when the interviews were conducted. While respondents were not asked about their own experiences of violence, we followed best practices for research with women and girls who experience violence (54) by only conducting the interviews when respondent privacy was assured and by ensuring that the interviewer was trained in how to conduct research related to VAWC, including listening without judgement. We were not able to offer psychological counselling for midwives who wanted to process the violence they had witnessed or heard about because we were not able to secure funding for psychosocial support services, which were not publicly available given that we did not have funding for this work.

## 4 Results

The findings from the interviews were coded into 27 distinct codes, which are detailed in Supplementary Table S2. These codes were systematically grouped into three overarching themes. The first theme focused on midwives' demographic characteristics and training. The second theme explored midwives' experiences in responding to VAWG, along with the barriers they faced in addressing these issues. The third theme highlighted available resources outside survivors' families, as well as the facilitators and barriers associated with accessing these resources.

### 4.1 Midwives' characteristics and backgrounds

**Figure 1:**
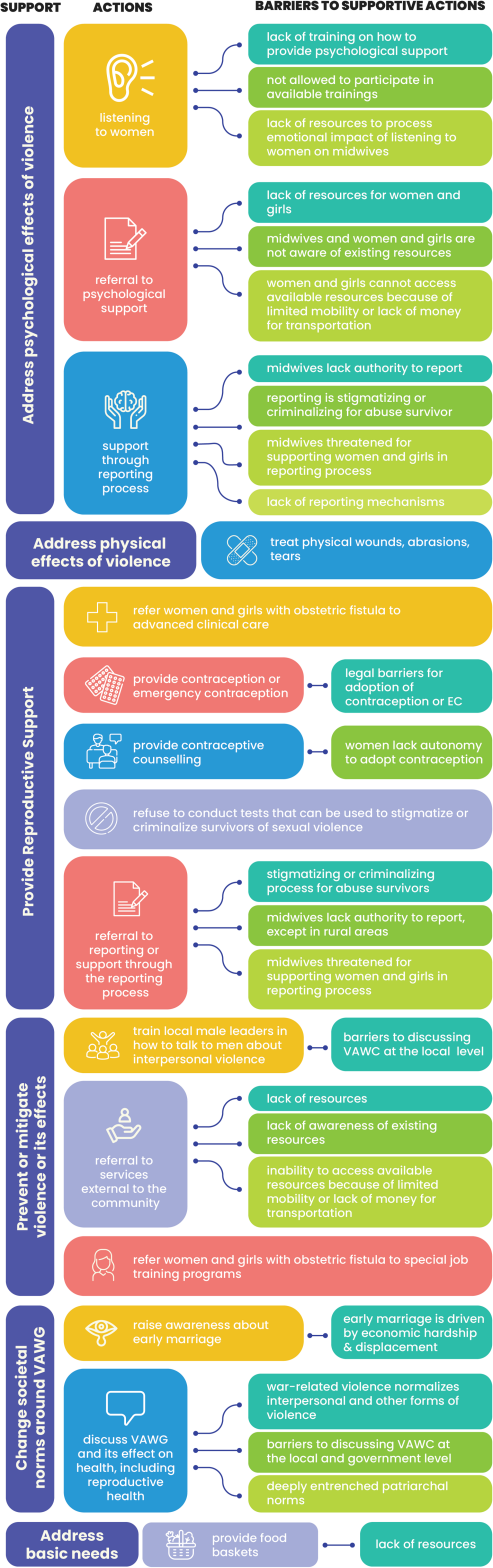
Midwives' response and barriers addressing VAWG.

Table 1 provides basic demographic information for study participants. Half of midwives (*N* = 10, 50%) were between 30 and 39; seven (35%) were between 40 and 49, and very few (*N* = 3, 15%) were under 30. Most (*N* = 14, 70%) midwives were married; four (20%) were single; one was divorced, and one was widowed. Nearly half of the midwives (*N* = 11, 55%) had 1–5 children, three (19%) had more than five children and three had no children. Almost half of the midwives (*N* = 11, 55%) were classified as community midwives. This includes midwives who had completed 9 years of elementary education, followed by three years of midwifery training (55). 20% (*N* = 4) were technical midwives who had completed 12th grade of formal education, followed by a diploma in midwifery (55). One quarter (25%) were midwifery trainers. All midwives had worked in their profession for over ten years. Most participants worked in urban (*N* = 12, 60%) vs. rural (*N* = 8, 40%) areas. Close to half (*N* = 9; 45%) worked in hospitals, 35% (*N* = 7) in health centres or small health units, and 15% (*N* = 3) in the Ministry of Health or its district office. One midwife worked in a private clinic. We summarise the role of midwives in supporting women and girls who experience violence and barriers to that work in Figure 1.

**Table 1:** Demographic characteristics of interview participants (*N* = 20).

Characteristics	*N*	%
Interview modality		
Face to-Face	15	75%
Over the phone	5	15%
Age (years)		
30-39	10	50%
40-49	7	35%
50 or older	3	15%
Marital status		
Single	4	20%
Married	14	70%
Widow	1	5%
Divorced	1	5%
No. of children		
No children	3	19%
1-5 children	10	63%
>5 children	3	19%
Midwives' state of origin		
Sana'a Amantasmiah	2	10%
Sana'a Government	5	25%
Taiz	7	35%
Aden	5	25%
Ibb	1	5%
Midwives primary work location		
Sana'a Amanat Al-Asimah	5	25%
Sana'a Governorate	5	25%
Taiz	5	25%
Aden	5	25%
Certification level		
Community midwife (intermediate diploma)	11	55%
Professional Midwife (high diploma)	4	20%
Midwife trainer	5	25%
Number of years in the profession		
Up to 10 years	0	0%
Over 10 years	20	100%
Where the midwives work		
Hospitals	9	45%
Health facility	6	30%
GHO/DHO	3	15%
NYMA	1	5%
Private clinic	1	5%
Population served		
Urban	12	60%
Rural	8	40%

GHO, governorate health office; DHO, district health office; NYMA, national yemeni midwife association.

### 4.2 Training on VAWG identification and response

About half of midwives (*N* = 9, 45%) had never received any VAWG-related training. Midwives who had received training said they had learned clinical management of victims of sexual violence through the Minimum Initial Service Package (MISP) (56) women's SRH, or other women's empowerment-related training. Midwives reported that they had received minimal training on VAWG-specific issues because of cultural sensitivities, stigma, and societal violence related to discussing or otherwise addressing VAWG, including social and legal challenges.

A long time ago, I received training related to early marriage, but these types of training are prohibited because of the customs and traditions. Because we are a tribal people, the law is almost non-existent, and the ruling comes from sheikhs and other leaders based on the customs and traditions. (MW-04, Amanat Al-Asimah, urban, aged 30–39)

I was recently asked to attend training on women's empowerment, but I declined. If the ministry allowed such training, I would have attended. The Ministry of Health doesn't allow this training because society rejects the idea of such programs (MW-04, Amanat Al-Asimah, urban, aged 30–39)

### 4.3 Midwives' response to VAWG

Women and girls often turn to midwives for treatment and support related to the psychological, physical, and reproductive sequelae of violence they have experienced.

#### 4.3.1 Provide psychological support

Midwives frequently provide psychological support, which is the most common form of care they offer to survivors of violence. Women experiencing emotional abuse often share their stories with midwives during visits for help after abuse or when they attend SRH services. By creating a safe and confidential environment, midwives build trust and provide reassurance, encouraging survivors to openly discuss their experiences. “I absorb her anger and talk to her.” (MW-18, Aden, urban, aged 30–39), reported one midwife about her initial response to abused women. Another midwife shared, “*I provide psychological support to the women who come to my clinic for help. One woman told me, ‘I feel comfortable when I talk to you.”* (MW-11, Taiz, rural, aged 30–39).

#### 4.3.2 Address physical and reproductive health-related consequences

Midwives play a critical role in addressing the physical consequences of violence. Many shared cases where they treated injuries from physical abuse, including beatings by intimate partners. One participant shared “Once, a runaway girl came to me, asking me to clean her wounds caused by her husband's beating. She was in a terrible situation.” (MW-03, Amanat Al-Asimah, urban, aged 30–39).

Midwives play a critical role in providing care and support for women and girls who experience sexual violence, including marital rape. Their contributions are particularly significant in addressing the health consequences of early marriage and sexual violence, including unwanted pregnancy, vaginal tears, bleeding, obstetric fistula, and spontaneous miscarriage. This includes providing immediate healthcare, documenting cases when necessary, and referring survivors to specialized services, such as obstetricians or PEP services.

I saw some cases complaining of violence from their husbands during sex -forced or violent sex, which also includes beating. Others come to us because of bleeding or tears. (MW-16, Aden, urban, aged 40–49)

In the few incidents of sexual violence reported to midwives, they provided empirical emergency contraception to women and girls following sexual violence to prevent pregnancy.

The mother had a disabled child whom she regularly took to the 22nd of May Hospital in Haziz for physiotherapy. So, her daughter was at home alone. While the daughter was collecting prayer clothes in the yard, someone raped her. I gave her eight contraceptive pills because I was afraid the girl would get pregnant. (MW-03, Amanat Al-Asimah, urban, aged 30–39)

Women or their families sought midwives' services to conduct a virginity test before marriage, after genital trauma or sexual assault. Several midwives reported instances where they refuse to conduct such a test to avoid conflicts between families and protect women.

Some women only want to have their hymen checked following sexual assault, but I refuse and refer them to the hospital. (MW-18, Aden, urban, aged 30–39)

Once, a bride came to me on her wedding night. The families of the bride and groom were all armed. They wanted me to allow the bride's mother to enter the examination room with us. When I saw this situation, I was terrified and refused to expose the girl and disclose what I saw. Instead, I explained that I was not authorized to provide such a report. As midwives, we often keep information confidential for the safety of families. (MW-07, Amanat Al-Asimah, urban, aged 40–49)

#### 4.3.3 Mitigate the effects of VAWG

Midwives play a critical role in mitigating the effects of violence by empowering women and linking them to additional health services. These include referrals to hotlines, women-friendly spaces (WFSs), and shelters where survivors can access legal services, economic grants, food baskets, or participate in empowerment initiatives and projects available within these facilities.

If we receive an abused woman, we refer her to Al-Sadaka Hospital. They have a dedicated department for victims of violence, where cases are received, and medical and psychological support is provided. (MW-20, Aden, urban, aged 40–49)

I refer abused women usually to the support programs, which are provided for example by Yemeni Women's Union or other partners. (MW-01, Amanat Al-Asimah, urban, aged 40–49).

All participants recognized obstetric fistula as a severe consequence of early marriage and related obstetric complications. Women who suffer from obstetric fistula often face profound social challenges, including isolation, depression, and rejection by partners or family members who view them as a burden.

Midwives play a pivotal role in identifying women affected by obstetric fistula. They provide essential information about the condition and refer these women to supported hospitals, where they can access free surgical treatment. After receiving treatment, women are referred to women-friendly spaces (WFSs), where they are offered vocational training programs to support their livelihood and rebuild their lives.

We have a representative midwife in each governorate. When a woman with obstetric fistula arrives, we report the case to the supported hospital so that she can receive a treatment card and travel allowance. (MW-01, Amanat Al-Asimah, urban, aged 40–49)

#### 4.3.4 Change societal norms around VAWG

Midwives can significantly contribute to changing community norms and attitudes that perpetuate violence by raising awareness about key issues such as early marriage, access to family planning, sexual education, and personal hygiene.

We raise awareness about early marriage every Thursday, and we also assist pregnant women. Girls, even if they reach puberty, are not ready for marriage. Marriage is a responsibility and requires maturity. (MW-08, Sana'a Gov, rural, aged 30–39)

Midwives are recognized as trusted figures within their communities, and couples often turn to them for conflict resolution and counseling.

Once, a woman came to me, she had been severely abused by her husband, who had attacked her multiple times. She was his third wife. I provided her with treatment and helped resolve the issue between them. (MW-11, Taiz, rural, aged 30–39)

Midwives not only provide healthcare but also play a key role in addressing violence by collaborating with community leaders (Shiekh). In some cases, they report incidents of abuse to ensure the safety and well-being of those affected.

… there is a woman whose husband beat her and divorced her. The woman resorted to the neighborhood Sheikh, who referred her to me to check if she was abused, so the Sheikh took her for a day and ordered the husband to leave the house for her and her children. (MW-08, Sana'a Gov, rural, aged 30–39)

A case came to me about a woman who was physically abused by her husband, which caused bleeding and a miscarriage. My husband yelled at me not to interfere, but I convinced him to report the matter to the neighborhood sheikh. The sheikh spoke to the man and took action. Usually, the sheikh educates the husband, but if that doesn't work, we report it to the local authorities. (MW-08, Sana'a Gov, rural, aged 30–39)

### 4.4 Barriers to supporting women and girls who experience violence

Midwives reported numerous barriers to providing care for survivors of VAWG, including insufficient training, a lack of authorisation, and the absence of comprehensive VAWG-specific practice guidelines. A midwife reported “Doctors are the ones who examine the case.” (MW-04, Amanat Al-Asimah, urban, aged 30–39), and two others said, “I refer the case to a specialist.” (MW-09, Sana'a Gov, rural, aged 30–39), “I do not conduct the physical examination following sexual assault because we lack the authority” (MW-18, Aden, urban, aged 30–39).

If the doctor is present in the hospital, she will be responsible for the case, not the midwife. This is because rape cases require a referral to the police and a report from the doctor. Therefore, we only guide the survivor. (MW-16, Aden, urban, aged over 50)

Midwives also highlighted the role of violence-associated stigma and gender-inequitable norms in limiting the options available to women and girls who experience violence.

If a woman experiences violence, she may not go to the hospital due to inhumane treatment, so women often choose silence over facing humiliation. (MW-07, Amanat Al-Asimah, urban, aged 40–49)

In seeking to support women who experience violence, midwives referred to the importance of respecting women's autonomy to make decisions about reporting and care.

We cannot act without a woman's consent. But we advise her to speak up and report, and we ask her if she wants our help so that we can report and support her. But if she refuses, we cannot force her; everything is optional*.* (MW-2, Amanat Al-Asimah, urban, aged 39–40)

Midwives felt that they should be trained to support women and girls experiencing violence.

As midwives, we must know how to provide psychological support, where to refer abused women or children, and what services are available. (MW-13, Taiz, urban, aged 40–49)

Midwives felt that they could not directly address VAWG outside of providing emotional support, medical assistance, or referrals to specialized care because of their limited experience and training. Several midwives reported being threatened by the family or by officials for directly confronting violence or reporting the violence. A midwife mentioned “I don't intervene in these issues because I fear for my own safety and because I do not have the authority to do so.” (MW-07, Sana'a Gov, rural, aged 40–49).

Midwives also reported that women were afraid for the midwives' safety if they helped them with reporting.

Interviewer: Have you faced any difficult situations at work?

Respondent: Yes, twice with the same man. The first time I helped his wife during childbirth; she had complications, but her husband was aggressive and threatened to harm me if anything went wrong with their son. The second time, when he was physically abusing his wife, I advised her to seek a solution. That time I refused to report the incident. I was afraid for my safety and that of my children. (MW-06, Sana'a Gov, rural, aged 30–39)

Other midwives complained about a lack of knowledge about resources, a midwife said, “I have never heard of these services before. There are no safe spaces for women here, maybe because I live in a small village.” (MW-06, Sana'a Gov, rural, aged 30–39).

Most midwives indicated that sexual assault survivors were afraid to seek assistance, especially within the health system, due to confidentiality and stigma-related concerns.

Women are afraid to expose their bodies or claim their rights if they have experienced violence. I believe that solutions must be found to help women overcome their fears. (MW-13, Taiz, urban, aged 40–49)

### 4.5 Mechanisms for reporting sexual violence within the health system

Reporting mechanisms are inconsistent and poorly defined across contexts and levels of health care. While prompt documentation is critical, particularly for injuries, hymenal tears, or evidence of penetration, as these signs can degrade or lose their forensic value if the survivor waits until reaching the reference hospital, midwives suggested that most women and girls who experience sexual violence would be reluctant to report this violence or formally document related injuries. Beyond fear and stigma, financial hardships and the cost and challenges of traveling to hospitals may also discourage victims from accessing timely services.

Interviews indicated that midwives are required to notify the health facility's administrative staff before receiving cases of abuse, with doctors typically taking over for further management. Consequently, midwives often prefer to refer these cases to hospitals where specialized healthcare professionals are better equipped to provide comprehensive care and intervention However, in rural areas or smaller health facilities in cities, midwives frequently serve as the primary healthcare providers. In such scenarios, they may be required—upon request from facility managers or community leaders (Sheikhs)—to document immediate signs of violence and support abused women.

In cases of sexual assault, I can take some notes when receiving the case. This is because some signs of rape may have disappeared by the time the survivors arrive for the forensic examination. (MW-07, Sana'a Gov, rural, aged 40–49)

In rural settings, midwives report incidents of violence to the hospital administrative staff, who may then alert the village's community leader. The leader may either address the issue locally or escalate it to the police.

In urban areas, midwives notify hospital administrative staff of the evidence for sexual assault. If the case proceeds, a committee of four doctors is then responsible for assessing and documenting the violence, including physical and sexual injuries, for judicial purposes.

When I receive the case, I take the medical history, then inform the doctor in the department, the administrative officer, and the security officer at the hospital. They, in turn, take the necessary legal actions and request the doctors to prepare a comprehensive report on the incident. After that, 3 to 4 doctors will examine the case and send the report to the responsible authority. (MW-04, Amanat Al-Asimah, Urban, aged 30–39)

Although midwives understand the formal procedures for reporting rape, they expressed concerns that formal procedures are infrequently followed due to survivors' fears, the stigma associated with reporting such incidents, and safety concerns for both the midwives and survivors. Midwives in rural areas or at small facilities in urban areas have to refer women or girls who report sexual violence to larger hospitals, sometimes outside their cities. However, women and girls are generally not able to travel to these larger hospitals due to mobility restrictions, financial barriers, and societal stigma.

### 4.6 Resources available for women and girls who experience violence that is external to the family

Respondents identified women-friendly spaces as an essential resource for VAWG survivors. These spaces provide access to information and psychological care for VAWG survivors and help survivors rebuild their networks and strengthen their relationships with peers. Women in these spaces learn sewing, embroidery, and incense production, which they can sell to support themselves. They can apply to receive limited funding to start their independent businesses, e.g., funding for a sewing machine.

There is an organization in my region that supports abused women by teaching women sewing, embroidery, and incense production. These services were provided at health centers. (MW-11, Taiz, rural, aged 30–39)

Participants listed the Yemeni Women's Union (YWU) and other empowerment-related organizations as resources for women and girls who experience violence. Midwives felt that YWU shelters could offer women a safe and secure environment to recover from their experiences and receive psychological support, education, vocational training, and job opportunities.

I know that the Yemeni Women Union assists women who experience violence. They have a special department to receive, provide psychological support, and refer them to other medical and legal services. (MW-20, Aden, urban, aged 40–49)

Midwives also described the importance of legal support from the YMU or other groups, which may provide free legal assistance, including court representation, counseling, and assistance in filing police reports and pressing charges against perpetrators.

There are initiatives by Yemeni lawyers to help women who want a divorce and also provide them with a safe life. (MW-09, Sana'a Gov, rural, aged 30–39)

Several midwives reported that YWU, in collaboration with other NGOs, offers a helpline and shelter services to women and girls who have been victims of violence. Some midwives described the hotline as staffed by trained professionals who can provide information, counseling, and referrals to other resources such as shelters and legal assistance. Although midwives were not confident about the services provided by the hotline, those who knew the number shared it with survivors.

There is a hotline supported by Marie Stopes. If a woman wants to inquire about a case of rape or an unsafe pregnancy, not only limited to violence experienced by her husband, she can report her case online for free. (MW-02, Amanat Al-Asimah, urban, aged 30–39*)*

Midwives said that women or girls receiving obstetric fistula treatment could also be referred to a special women's empowerment program that includes education and job development programming. While most midwives could name at least one service to which women could be referred for legal support or shelter, most midwives said they did not have enough information on VAWG-related services. Midwives from rural areas reported no local services beyond the Sheikh or the woman or girls' family.

I have never heard of these services before. There are no safe spaces for women here, maybe because I live in a small village. (MW-06, Sana'a Gov, rural, aged 30–39)

While midwives were able to name services for women who experienced violence, they did not know of any women who had used the services and doubted whether the services were accessible to women and girls.

Interviewer: Have you reported or referred a battered woman to the hotline?
Respondent: No one would call; women are afraid to report.
Interviewer: How are they helped by the hotline?
Respondent: It only provides counselling.
Interviewer: Are they referred to hospitals?

Respondent: I don't know exactly what they do, but this hotline provides services and counselling, as I have heard…I don't know if anyone has benefited or not, because most of the cases disappear after the first time we see them. (MW-02, Amanat Al-Asimah, urban, aged 30–39).

### 4.7 Barriers to accessing VAWG services

Midwives identified multiple factors that prevent women and girls from accessing VAWG-related services, including social stigma, limited knowledge about available resources, and a lack of trust in available services. In some cases, traditional values and beliefs contributed to instances where women who reported VAWG could be themselves imprisoned or otherwise socially and economically isolated. In addition, the pervasive social stigma associated with seeking protection services and the deep fears of survivors and their families of being identified or stigmatized are significant barriers that prevent women and their families from reporting or accessing resources within their communities (e.g., local Sheikh).

Women are afraid to expose their bodies or claim their rights if they have experienced violence. I believe that solutions must be found to help women overcome their fears. (MW-13, Taiz, urban, aged 40–49).

Respondents reported that VAWG services were cut off or otherwise limited due to the ongoing humanitarian crisis, the subsequent COVID-19 pandemic-associated shifts in health priorities, and political reasons.

In war-torn countries, addressing violence is considered less important than saving lives. This is the perspective of humanitarian organizations. As a result, support is primarily limited to emergency response. (MW-01, Amanat Al-Asimah, urban, aged 40–49)

Midwives also said that they did not think most women or girls had ever heard about existing VAWG services.

Look, an ordinary person cannot access these services, so organizations should promote and advertise these services. If we do statistics on the extent of women's knowledge of the services provided by the Yemeni Women's Union, the percentage will not exceed 10%. (MW-07, Amanat Al-Asimah, urban, aged 40–49).

Midwives reported that sexual violence is an especially sensitive topic for midwives and women who experience violence since women who become pregnant following sexual violence can be jailed. This risk can discourage survivors from reporting or accessing essential services.

We have a Criminal Investigation Department in the hospital; if there are cases of sexual violence, or if a woman is suspected of being pregnant without being married, they take her to prison. (MW-15, Taiz, urban, aged 30–39)

VAWG-related interventions aimed at addressing violence and supporting women confront significant opposition from governmental authorities, community leaders, and the served population.

Any course related to gender-based violence requires a complex process through the Ministry of Health to be implemented because society does not accept these things. (MW-02, Amanat Al-Asimah, urban, age 25–34)

Survivors of sexual violence often face disrespectful and inadequate treatment within healthcare facilities. This judgmental attitude discourages women from accessing essential support available for survivors of sexual violence including emergency contraceptives.

If a woman experiences violence, she often avoids seeking help at hospitals due to the inhumane treatment she may receive. Consequently, women frequently choose to remain silent rather than endure further humiliation. (MW-07, Amanat Al-Asimah, urban, age 35 or over)

## 5 Discussion

We found that community midwives play an essential role in addressing violence-related complications, including providing psychological support, raising awareness about the causes and consequences of violence, working with local Sheiks to support women and girls who experience violence, and offering preventative care, like emergency contraception, to women and girls at risk of experiencing sexual violence. Midwives enumerated several important barriers and risks associated with their work on VAWG-related issues, including threats to women for engaging in reporting, threats to themselves for supporting women in the reporting process, and difficulty in participating in training related to women's empowerment, VAWG, and SRHR because of the stigma associated with discussing VAWG. Most midwives did not have sufficient information about available resources or confidence that resources would be accessible or could help women. Midwives reported that the stigma and violence associated with reporting or accessing services through the formal health system were a more significant threat than the violence itself, which highlights the importance of midwives' role in supporting women and girls who experience violence during their home visits. Midwives used all available resources to support women and girls who experienced violence, including refusing to conduct procedures like assessing whether the hymen was intact following sexual violence, which could be used to criminalize women and girls who experience sexual violence outside of marriage. Their expertise and ability to work within local constraints highlight the need for financial support for locally developed approaches to identifying, preventing, and mitigating violence.

In keeping with the WHO guidelines that advise against mandatory reporting of VAWG (57), because of safety-related concerns, midwives felt women and girls should make their own decisions about reporting or addressing the violence. This finding aligns with other studies and recommendations that specify that survivors should decide when or whether to report their exposure to violence and who to report to (58). Midwives reported that women and girls infrequently reported sexual violence, which is similar to other studies that found that sexual violence is highly stigmatized and often goes unreported (59–61) and with findings from a recent study in Morocco, where patriarchal societal norms were also associated with underreporting of sexual violence (62). While midwives in Yemen are not eligible to prepare comprehensive medical documentation, they are permitted to create a preliminary report in specific situations, such as the unavailability of a physician or in a rural setting, to document any sign of sexual assault, that might otherwise be overlooked during referral process to a reference hospital. Midwives said that emotional support was the most common way they supported women and girls who experienced violence, which aligns with findings from a systematic review of qualitative studies that found that healthcare professionals' primary response to support survivors of VAWG was to offer emotional support and information on resources (63).

The main barriers to midwives supporting women and girls who experience VAWG were the absence of guidelines or training, insufficient resources both within and outside of local communities, societal norms and policies that enable VAWG or restrict survivors' ability to respond to VAWG, and threats to midwives from perpetrators, survivors' families, or from other community sources, including law enforcement. These findings correlate with a systematic review of qualitative studies with health care providers on VAWG and related studies in other contexts (26, 63, 64) and a recent qualitative study that found that threats to their own safety from perpetrators were an important barrier for Swedish midwives addressing VAWG in the context of SRHR (64). In keeping with other qualitative research with nurses and other health workers (58–61, 65), some participants in this study felt that addressing VAWG was outside their role as midwives. As highlighted here, research in other contexts has identified stigma associated with VAWG and fears of increased violence, divorce, or losing custody of children as important barriers to reporting and accessing services (26, 60, 64), suggesting that these are global rather than local challenges.

WHO released a 2013 guideline summarising existing evidence and highlighted the importance of healthcare providers in identifying VAWG and supporting survivors. VAWG-related training for health professionals has been shown to enhance referrals to external services (57). Our finding that midwives have not received formal training to support women and girls who experience violence is supported by several other studies that have found that midwives feel inadequately prepared to respond to VAWG (26, 58). All midwives reported the need for further training to better support women and girls who experience violence. Studies in other LMICs found health professionals reported inadequate training on VAWG and a lack of integration of VAWG-related subjects into the health professionals' standard curricula. Outside of the healthcare sector, the studies have identified a general lack of training opportunities, guidelines, and funding for addressing VAWG (26, 58, 64, 66).

While VAWG increases during conflicts and pandemics, support for Yemeni women and girls who experience violence, including shelters and vocational training, decreased during the war and the COVID-19 pandemic (48).

A study in Bangladesh evaluating women's empowerment programs showed that women who participated in these livelihood programs were less likely to experience IPV (59). While any interventions need to be supported by formative research to understand the local context and develop safety plans and safely accessible local resources to support women and girls who experience violence, providing support for basic needs and developing community-led women's empowerment programs could be one way to support Yemeni women and girls who experience violence. Ensuring community midwives can better support women and girls who experience violence and funding community-led work to address gender inequities aligns with recommendations from a recent situation report in Yemen that stated that women's protection services, including women's shelters and local capacity in the health and protection sectors should be supported through funding and resource mobilization (34).

### 5.1 Strengths and limitations of this study

This study has several strengths. This formative study is the first to assess the practices and preparedness of the Yemeni community midwives to respond to VAWG. We identified several important barriers to midwives' work, including policymakers' lack of support for their participation in training that relates to violence and their receipt of threats when reporting violence experienced by the women or girls they serve. Findings from this study can inform the development of safety planning materials for midwives and the women and girls they serve and curricula to safely support midwives' work with women and girls who experience violence. This study provides an initial first step towards understanding the work needed to develop safety planning materials, evidence-based guidance, and capacity building for midwives to support GBV survivors better. NYMA reviewed the study protocol and consent form to ensure they were context-appropriate. This study has some limitations. Although saturation was reached, a larger sample, where other governorates can be included, is warranted. Ensuring the safety of the research team and participants in a protracted humanitarian crisis was further complicated by the COVID-19 pandemic, and some interviews were conducted over the phone due to safety concerns. We were not able to secure funding to support this work. There were no available mental health services for research participants, and we could not offer counseling for midwives who would have liked to process their experience working with women and girls who experience violence with a mental health professional because of the lack of funding.

### 5.2 Next steps for supporting midwives' work with women who experience violence

In Yemen, where cultural values and beliefs may prevent women and girls from interacting with the formal health system and from reporting violence, community midwives are a critical resource for women and girls who experience family violence. Midwives are often the first point of contact with the health system for women and girls who experience violence and should be trained and equipped to support women and girls who experience violence (57, 67).

Yemeni women and girls experience significant barriers to accessing VAWG services, and Yemeni midwives face similar barriers to reporting VAWG that they observe or are told about. This formative research is the first step to developing an enabling environment for research related to SRH in the context of VAWG in a humanitarian setting where both SRH and VAWG are highly stigmatised. A comprehensive understanding of the context and consequences of research and programs meant to identify and mitigate VAWG is essential. Midwives and the women and girls they serve should be at the forefront of that work to ensure the safety of women and girls who experience violence and the health professionals, like community midwives, who try to support them. While integrating care for VAWG- survivors into health services is highly recommended to remove the stigma related to accessing services, the health system's preparedness should first be enhanced to serve battered women and girls (57, 68).

## 6 Conclusion

The ongoing war in Yemen has set back meaningful advances in addressing child marriage, gender equality and reduced the resources available for women and girls to avoid, mitigate, confront, or otherwise address the violence in their lives, underscoring the need to understand and address VAWG in Yemen. Midwives come from and live in the communities that they serve and are well-placed to engage women and girls in conversations about family violence and in thinking through how violence can be measured, prevented, and addressed. While some materials have been developed to train healthcare workers on how to provide psychosocial and immediate care to women and girls who experience violence, these materials need to be adapted for Yemen, adopted, and integrated into midwifery education and services (49, 62). Midwives play a crucial role in supporting women and girls who experience violence in Yemen. NYMA-trained community midwives are respected members of their localities and serve as an essential, informal resource for women and girls who experience violence. Emphasizing the importance of building the capacity of community midwives and supporting their work in areas of SRH/VAWG to effectively empower abused women and girls should be considered central to advancing the global development agenda. Midwives and local Sheikhs were trusted resources for women and girls who experienced VAWG. Subsequent research could explore how to build collaboration between midwives and local Sheikhs to strengthen support for women and girls who experience violence.

Established cultural norms and violence-related stigma constitute barriers to the disclosure and reporting of violence, further restricting access to the available health and protective services. Ensuring the allocation of funding for VAWG interventions that focus on identifying and addressing VAWG in Yemen while advocating for community-oriented initiatives ought to be a key focus for international donors who are committed to enhancing MCH, the SRHR of women and girls, and the associated Sustainable Development Goals (SDGs).

## Data Availability

The raw data supporting the conclusions of this article will be made available by the authors, without undue reservation.
