# Supplementary material for: Midwives' experiences working with women and girls surviving violence in Yemen: a qualitative study
Source: Front Glob Womens Health. 2025 Mar 21;6:1450053. doi: 10.3389/fgwh.2025.1450053 (PMC11968663; doi:10.3389/fgwh.2025.1450053)
Supplement: Supplementary file 1 [file Table1.docx]

Supplementary Material

# Supplementary Figures and Tables

## Supplementary Tables

**Supplementary Table S1**. Ovid(Medline) Search strategy

| 1 | exp Violence/ |
| --- | --- |
| 2 | (violence adj3 (women or girls or children)).ti |
| 3 | (child adj3 (labor or labor or marriage or soldier)).ti |
| 4 | ((gender*based or intimate partner or domestic or honor or honour or school or war or conflict or pandemic or COVID) adj2 violence).ti |
| 5 | (traffic* or tourist marriage or capital punishment or corporal punishment).ti |
| 6 | Midwifery/ |
| 7 | (midwife or midwiv* or community health worker*).ti |
| 8 | 1 or 2 or 3 or 4 or 5 |
| 9 | 6 or 7 |
| 10 | 8 and 9 |
| 11 | Limit 10 to yr=”2019-Current”* |
| 12 | Yemen/ |
| 13 | Yemen.mp |
| 14 | 12 or 13 |
| 15 | 11 and 14^+^ |

*yielded 184 citations; ^+^yielded 0 citations

**Supplementary Table S2. Codebook**

| **Code in MAXQDA** | **Definition** |
| --- | --- |
| 1. VAWG – Description | Type, frequency, perpetrator, location or consequences of violence against women |
| 1. VAC – Description | Type, frequency, perpetrator, location or consequences of violence against children |
| 1. Violence in pregnancy | Any mention of violence in pregnancy, including perpetrator, type of violence, frequency of violence, services available, reporting violence against pregnant women, reasons for not leaving during pregnancy, any mention of violence in pregnancy |
| 1. Reasons for violence | What can be the roots or reasons of violence. |
| 1. VAWG - External services/support | This is the support outside the survivor’s family. The services for women exposed to violence (e.g., financial assistance, food basket, shelter, referral services, psychological support, legal actions, protection services). Anything related to family planning or obstetric fistula services would be included here. |
| 1. VAC - External services/support | Services and support for children exposed to violence, including referral, psychological support, protection service, shelter service |
| 1. VAWG - family resources/support | What resources are available to women to prevent or mitigate the violence that they experience that they have within themselves or their families |
| 1. VAC - family resources/support | What resources are available to children to prevent or mitigate the violence that they experience that they have within themselves or their families |
| 1. VAWC - Impact of war/COVID-19 on type of violence | How are the types, perpetrators, and frequency of violence experienced differently because of COVID-19 or the war? |
| 1. VAWC - Impact of war/COVID-19 on services | How are the types of services or access to services different because of COVID-19 or the war |
| 1. VAWC - Rec for improving services | Recommended that is given by midwives/stakeholders to improve the services for women or children exposed to violence (more health services, access to education or other services, transportation to services) |
| 1. Services - Refusal or inability to access | Reasons for refusal or inability to access available services, including shelter, mental health care, financial support |
| 1. Shelter - Restrictions in access | Lack of transportation, need for consent from male family members, etc. |
| 1. Shelter - Restrictions during the stay | How long women who have/have no kids can stay in the shelters (less, more, or no change than those who have no children; can children accompany their mothers? How many children can accompany their mother; can't go outside alone, need to go back early, can't meet family...etc) |
| 1. Shelters - preparing Women & Children to return | Education, skill building, job training, pocket money, assistance for women or children (e.g.., financial, transportation, follow-up, mental health support, family therapy) when they return to their family |
| 1. Shelter - services or financial support | Education, skill building, job training, pocket money |
| 1. Midwives/stakeholder response to violence against women/children | Midwives report, midwives do not report, how midwives decide whether to report or not report, including taking permission from the woman or child who experiences violence, whom midwives report to |
| 1. Women/children response to violence | What are the measures taking by women to reduce the violence that women exposed to (e.g ask help from family, freind, leave home, accept or reject violence,not react) |
| 1. Consequences of confronting/reporting violence against women/children for midwives/stakeholders | How midwives/stakeholders are affected when they observe/confront /offer help or report on violence against women or children. |
| 1. Participant's own - witnessing or experiencing VAWG | Participant's own experience witnessing or experiencing violence against women, including violence against themselves |
| 1. Participant's own - witnessing or experiencing VAC | Participant's own experience witnessing or experiencing violence against children, including violence against themselves as children |
| 1. VAWG - why are no services outside of the family | The reasons that NGOS or INGOS don't work in the women's protection sector( lack of funds, limited accessibility to some areas, rejection of these projects) |
| 1. VAC - why are no services outside of the family | The reasons that NGOS or INGOS don't provide services for children who experience violence ( lack of funds, limited accessibility to some areas, rejection of these projects) |
| 1. Recommendations - mitigate VAWC | The role that different actors play in reducing the violence that women or children are exposed to, including community, NGOs, Yemeni government, and tribal system. |
| 1. Training - VAWC | Any reference to training related to violence against women or children, including training duration, provider, quality, content (e.g. safety planning, counselling, appropriate language), need for more training |
| 1. Services - for exposures other than VAWC | Include description of services for IDPs |
| 1. Good quote | Any quote that represents very well one of the above points |

VAWG: violence against women and children, VAC: violence against children, VAWC: violence against women and chilren

**Supplementary Table S3.** Themes related to the role of midwives in addressing VAWC and representative participant quotes.

| **Theme** | **Representative Quote (English)** | **Representative Quote (Arabic)** |
| --- | --- | --- |
| **Capacity building** | | |
| (MW-04, Amantasimah, urban, aged 30-39) | "A long time ago, I received training related to early marriage, but these types of training are prohibited because of the customs and traditions. Because we are a tribal people, the law is almost non-existent, and the ruling comes from sheikhs and other leaders based on the customs and traditions”. | "منذ وقت طويل تلقيت تدريب عن الزواج المبكر، هذا النوع من التدريبات غير مسموح بها بسبب العادات والتقاليد. نحن مجتمع قبلي، القوانين تكاد تكون معدومة ولذلك التحكيم يأتي من الشيوخ والحكام ويكون مبني على العادات والتقاليد. " |
| (MW-04, Amantasimah, urban, aged 30-39) | " I was recently asked to attend a training called 'Women Empowerment', but I refused. If the Ministry allowed such training materials, I would have been able to attend, but the Ministry prohibits such training because the community rejects the idea of such training materials. If the ministry allowed such training materials, I would have been able to attend, but the ministry forbids it because the community rejects the idea of such training materials." | "طلب مني منذ قريب ان احضر تدريب عن تمكين المراءة، ولكني اعتذرت. إذا الوزارة تسمح بمثل هذه التدريبات كنت سأحضر. وزارة الصحة تمنع هذه التدريبات لأن المجتمع يرفض فكرة هذه التدريبات. " |
| **Midwives’ response in VAWG** | | |
| MW-18, Aden, urban, aged 30-39) | “I absorb her anger and talk to her.” | "أمتص غضبها وأتحدث إليها". |
| (MW-11, Taiz, rural, aged 30-39) | "I provide psychological support to the women who come to my clinic for help. One woman told me, 'I feel comfortable when I talk to you." | "أقدم الدعم النفسي للنساء اللاتي يأتين إلى عيادتي بحثًا عن المساعدة. مرة قالت لي امرأة: "أجد الراحة عندما أتي إليك للحديث". |
| (MW-03, Amantasmiah, urban, aged 30-39) | “Once, a runaway girl came to me, asking me to clean her wounds caused by her husband’s beating. She was in a terrible situation.” | "في مرة آتت الي فتاة هاربة، ارادت مني تنظيف جروحها الناتجة من ضرب زوجها لها. كانت في حالة فظيعة." |
| (MW-16, Aden, Urban, aged over 50) | “I saw some cases complaining of violence from their husbands during sex -forced or violent sex, which also includes beating. Others come to us because of bleeding or tears.” | “رأيت بعض الحالات تشتكي من العنف من أزواجهن أثناء الجنس القسري أو الجنس العنيف "الذي يشمل أيضًا الضرب يأتي البعض الآخر إلينا بسبب النزف أو التمزق.” |
| (MW-03, Amantasmiah, urban, aged 30-39) | ….“ The mother had a disabled child whom she regularly took to the 22nd of May Hospital in Haziz for physiotherapy. So, her daughter was at home alone. While the daughter was collecting prayer clothes in the yard, someone raped her. I gave her eight contraceptive pills because I was afraid the girl would get pregnant.” | "...الأم كان لديها طفل معاق كانت تأخذه بانتظام إلى مستشفى ٢٢ مايو في حزيز للعلاج الطبيعي. لذلك، كانت ابنتها في المنزل وحدها. وبينما كانت الابنة تجمع ملابس الصلاة في الفناء، هاجمها شخص واغتصبها. قدمت لها ثمانية حبوب منع الحمل خوفاً من حدوث حمل للفتاة." |
| (MW-18, Aden, urban, aged 30-39) | Some women only want to have their hymen checked following sexual assault, but I refuse and refer them to the hospital. | "بعض النساء يرغبن فقط في فحص غشاء البكارة بعد التعرض للاعتداء الجنسي، ولكني أرفض ذلك وأحيلهن إلى المستشفى. " |
| (MW-07, Amanatasimah, urban, aged 40-49 | Once, a bride came to me on her wedding night. The families of the bride and groom were all armed. They wanted me to allow the bride's mother to enter the examination room with us. When I saw this situation, I was very afraid and refused to expose the girl and disclose what I saw. Instead, I explained that I was not authorized to provide such a report. As midwives, we often keep information confidential for the safety of families." | "مرة، جاءت عروس إلي في ليلة الزفاف. كانت عائلتا العروس والعريس مسلحتين. أرادوا مني السماح لأم العروس بدخول غرفة الفحص معنا. عندما رأيت هذا الموقف، خفت كثيرًا ورفضت فضح الفتاة والإفصاح عما رأيت. بدلاً من ذلك، شرحت أنني لا أملك الصلاحية لتقديم تقرير مثل هذا. كقابلة، نحن غالباً ما نحتفظ بالمعلومات سراً من أجل سلامة العائلات." |
| (MW-20, Aden, urban, aged 40-49) | “If we receive an abused woman, we refer her to Al-Sadaka Hospital They have a dedicated department for victims of violence, where cases are received, and medical and psychological support is provided.” | **"**إذا استقبلنا امرأة مُعَنّفة، فإننا نحيلها إلى مستشفى الصداقة. لديهم قسم مخصص لضحايا العنف، حيث يتم استقبال الحالات وتقديم الدعم الطبي والنفسي**."** |
| MW-01, Amanat Al Asimah, urban, aged 40-49) | “I refer abused women usually to the support programs, which are provided for example by Yemeni Women's Union or other partners” | "عادةً ما أحيل النساء المُعَنّفات إلى برامج الدعم اتقدمها، على سبيل المثال، الاتحاد النسائي اليمني أو أي شريك آخر". |
| (MW-01, Amanatasimah, urban, aged 40-49) | “We have a representative midwife in each governorate. When a woman with obstetric fistula arrives, we report the case to the supported hospital so that she can receive a treatment card and travel allowance.” | "لدينا قابلة في كل محافظة. عندما تصل امرأة تعاني من الفتق التوليدي، نقوم بالإبلاغ عن الحالة إلى المستشفى المدعومة حتى تحصل على بطاقة علاج وبدل سفر." |
| MW-08, Sana'a Gov, rural, aged 30-39). | “We raise awareness about early marriage every Thursday, and we also assist pregnant women. Girls, even if they reach puberty, are not ready for marriage. Marriage is a responsibility and requires maturity | "نقوم كل يوم خميس بالكثير من حملات التوعية عن الزواج المبكر بالإضافة الي دعم الحوامل. البنات حتى وان وصلت السن البلوغ فهي غير جاهزة لزواج. الزواج مسؤولية كبيرة وتحتاج الكثير من الوعي." |
| (MW-11, Taiz, rural, aged 30-39) | “Once , a woman came to me., she had been severely abused by her husband, who had attacked her multiple times. She was his third wife. I provided her with treatment and helped resolve the issue between them” | "جاءت امرأة إليّ. تعرضت لاعتداء شديد من زوجها، حيث هاجمها مرات عديدة. كانت هي زوجته الثالثة. قدمت لها العلاج وساعدت في حل المشكلة بينهما" |
| .(MW-08, Sana'a Gov, rural, aged 30-39) | “… there is a woman whose husband beat her and divorced her. The woman resorted to the neighborhood Sheikh, who referred her to me to check if she was abused, so the Sheikh took her for a day and ordered the husband to leave the house for her and her children | " هناك امرأة ضربها زوجها وطلقها. لجأت المرأة إلى شيخ الحي الذي أحالها إليّ للتأكد من تعرضها للإيذاء، فأخذها الشيخ لمدة يوم وأمر الزوج بمغادرة المنزل لها ولأولادها. " |
| ( MW-08, Sana'a Gov, rural, aged 30-39) | “A case came to me about a woman who was physically abused by her husband, which caused bleeding and a miscarriage. My husband yelled at me not to interfere, but I convinced him to report the matter to the neighborhood sheikh. The sheikh spoke to the man and took action. Usually, the sheikh educates the husband, but if that doesn’t work, we report it to the local authorities” | "ذات مرة، تعرضت امرأة للعنف الجسدي على يد زوجها، مما أدى إلى نزيف وإجهاض. صرخ زوجي في وجهي وأمرني بعدم التدخل، لكنني أقنعته بإبلاغ الشيخ في الحي بالحادثة. تحدث الشيخ مع الرجل واتخذ الإجراءات اللازمة. عادةً، يقوم الشيخ بتوعية الزوج، ولكن إذا لم تنجح تلك الطريقة، نقوم بالإبلاغ عن الحادثة للسلطات المحلية". |
| MW-13, Taiz, urban, aged 40-49) | “Women are afraid to expose their bodies or claim their rights if they have experienced violence. I believe that solutions must be found to help women overcome their fears.” | "النساء يخافن من الذهاب وكشف جسدهن أو استرجاع حقوقهن إذا تعرضن للعنف. أؤمن بأنه يجب البحث عن حلول لكي تستطيع النساء التغلب على مخاوفهن."\|. |
| (MW-15, Taiz, urban, aged 30-39) | We have a Criminal Investigation Department in the hospital; if there are cases of sexual violence, or if a woman is suspected of being pregnant without being married, they take her to prison. | "لدينا قسم للتحقيقات الجنائية في المستشفى؛ إذا كانت هناك حالات من العنف الجنسي، أو إذا كانت امرأة مشتبه في حملها بدون أن تكون متزوجة، يقومون بإيقافها في السجن.". |
| **Midwives’ barriers and challenges in supporting women and girls who experience violence** | | |
| (MW-04, Amanatasimah, urban, aged 30-39) | “Doctors are the ones who examine the case.” | "الأطباء هم الذين يفحصون الحالة." |
| (MW-09, Sana’a Gov, rural, aged 30-39) | “I refer the case to a specialist.” | "أحيل الحالة إلى اختصاصي." |
| (MW-18, Aden, urban, aged 30-39) | “I do not conduct the physical examination following sexual assault because we lack the authority.” | " أنا لا أُجري الفحص الجسدي بعد التعرض للاعتداء الجنسي لأنه لاتوجد لدينا صلاحيات." |
| (MW-16, Aden, urban, aged over 50) | “If the doctor is present in the hospital, she will be responsible for the case, not the midwife. This is because rape cases require a referral to the police and a report from the doctor. Therefore, we only guide the survivor.”. | "إذا كانت الطبيبة متواجدة في المستشفى ستكون هي المسؤولة عن الحال وليست القابلة. لأن حالات الاغتصاب تحتاج الي إحالة لشرطة وتقديم تقرير من الطبيب. لذلك نقوم نحن فقط بتوجيه الناجية." |
| (MW-07, Amanatasimah, urban, aged 40-49) | “If a woman experiences violence, she may not go to the hospital due to inhumane treatment, so women often choose silence over facing humiliation.” | "إذا تعرضت امرأة للعنف، قد لا تذهب إلى المستشفى بسبب المعاملة اللا إنسانية، لذا غالبًا ما تختار النساء الصمت على أن تتعرض للإهانة." |
| .(MW-2, Amanatasimah, urban, aged 39-40) | “We cannot act without a woman's consent. But we advise her to speak up and report, and we ask her if she wants our help so that we can report and support her. But if she refuses, we cannot force her; everything is optional.” | "لا يمكننا التصرف دون موافقة المرأة. ولكننا ننصحها بالتحدث والإبلاغ، ونسألها إذا كانت ترغب في مساعدتنا حتى نتمكن من الإبلاغ ودعمها. ولكن إذا رفضت، فلا يمكننا أن نجبرها؛ كل شيء اختياري. " |
| (MW-13, Taiz, urban, aged 40-49) | “As midwives, we must know how to provide psychological support, where to refer abused women or children, and what services are available.” | "كقابلات، يجب علينا أن نكون على علم بكيفية تقديم الدعم النفسي وإلى أين يمكن إحالة المرأة أو الطفل المعنّفين، وما هي الخدمات المتاحة." |
| (MW-07, Sana’a Gov, rural, aged 40-49) | “I don’t intervene in these issues because I fear for my own safety and because I do not have the authority to do so.” | "لا أتدخل في هذه المواضيع، لأني أخاف على سلامتي كما أنني لا أملك الكثير من الصلاحيات لتدخل". |
| (MW-06, Sana’a Gov, rural, aged 30-39) | Interviewer: Have you faced any difficult situations at work?  Respondent: Yes, twice with the same man. The first time I helped his wife during childbirth; she had complications, but her husband was aggressive and threatened to harm me if anything went wrong with their son. The second time, when he was physically abusing his wife, I advised her to seek a solution. That time I refused to report the incident. I was afraid for my safety and that of my children | “المحاور: هل واجهت أي مواقف صعبة في العمل؟  المُجيب: نعم، مرتين مع نفس الرجل. المرة الأولى، ساعدت زوجته أثناء الولادة؛ كانت تعاني من مضاعفات، لكن زوجها كان عدوانيًا وهددني بالإيذاء إذا حدث أي شيء سيء لابنهم. المرة الثانية، عندما كان يسيء معاملة زوجته جسديًا، نصحتها بالبحث عن حل. في تلك المرة، رفضت التبليغ عن الحادثة. كنت خائفًا على سلامتي وسلامة أطفالي. " |
| (MW-06, Sana’a Gov, rural, aged 30-39) | "I have never heard of these services before. There are no safe spaces for women here, maybe because I live in a small village." | "لم اسمع من قبل بهذه الخدمات. لا يوجد مساحات آمنة لنساء هنا ربما لأني أعيش في قرية صغيرة….." |
| . (MW-13, Taiz, urban, aged 40-49) | “Women are afraid to expose their bodies or claim their rights if they have experienced violence. I believe that solutions must be found to help women overcome their fears.” | "النساء يخافن من الذهاب وكشف جسدهن أو استرجاع حقوقهن إذا تعرضن للعنف. أؤمن بأنه يجب البحث عن حلول لكي تستطيع النساء التغلب على مخاوفهن." |
| (MW-15, Taiz, urban, aged 30-39) | “We have a Criminal Investigation Department in the hospital; if there are cases of sexual violence, or if a woman is suspected of being pregnant without being married, they take her to prison” | "لدينا قسم التحقيقات الجنائية في المستشفى؛ إذا كانت هناك حالات عنف جنسي، أو إذا كانت هناك امرأة يُشتبه في أنها حامل بدون زواج، تُؤخذ إلى السجن”. |
| **Mechanisms for reporting sexual violence within the health system** | | |
| (MW-07, Amanat Al Asimah, urban, aged 40-49) | “In cases of sexual assault, I can take some notes when receiving the case. This is because some signs of rape may have disappeared by the time the survivors arrive for the forensic examination.” | "في حالات الاعتداء الجنسي، يحق لي فقط تسجيل بعض الملاحظات عند استلام الحالة، لأن بعض علامات الاغتصاب قد تختفي بحلول الوقت الذي تصل فيه الناجيات إلى الفحص الشرعي". |
| (MW-04, Amanat al-Asimah , Urban, aged 30-39) | When I receive the case, I take the medical history, then inform the doctor in the department, the administrative officer, and the security officer at the hospital. They, in turn, take the necessary legal actions and request the doctors to prepare a comprehensive report on the incident. After that, three to four doctors will examine the case and send. | "بعد ان أستقبل الحالة، أقوم بأخذ القصة المرضية، ثم أبلغ الطبيب في القسم، والمسؤول الإداري، ومسؤول الأمن في المستشفى. هؤلاء بدورهم يتخذون الإجراءات القانونية اللازمة ويقدمون طلباً للأطباء لإعداد تقرير شامل عن الحالة. بعد ذلك، يقوم 3 إلى 4 أطباء بفحص الحالة وإرسال التقرير إلى الجهة المسؤولة". |
| **Resources available for women who experience violence** | | |
| (MW-11, Taiz, rural, aged 30-39 | “There is an organisation in my region that supports abused women by teaching women sewing, embroidery, and incense production. These services were provided at health centers.” | "هناك منظمة في منطقتي تدعم النساء المعنّفات من خلال تعليمهن الخياطة والتطريز وإنتاج البخور. كانت تقدم هذه الخدمات في المراكز الصحية.". |
| . (MW-20, Aden, urban, aged 40-49) | I know that the Yemeni Women Union assists women who experience violence. They have a special department to receive, provide psychological support, and refer them to other medical and legal services. | "أعلم أن اتحاد النساء يساعد النساء اللواتي يتعرضن للعنف. لديهم قسم منفصل لاستقبالهن وتقديم الدعم النفسي وإحالتهن إلى خدمات أخرى طبية وقضائية." |
| (MW-09, Sana'a Gov, rural, aged 30-39) | "There are initiatives by Yemeni lawyers to help women who want a divorce and also provide them with a safe life." | "هناك مبادرات من قبل محامين يمنيين (ناشطة يمنية) لمساعدة النساء اللواتي يرغبن في الطلاق وكذلك توفير حياة آمنة لهن." |
| (MW-02, Amantasmiah, urban, aged 30-39) | “There is a hotline supported by Marie Stopes. If a woman wants to inquire about a case of rape or an unsafe pregnancy, not only limited to violence experienced by her husband, she can also report her case online for free.” | "هناك خط ساخن مدعوم من قبل ماري ستوبس. إذا كانت امرأة ترغب في الاستفسار عن حالة اغتصاب أو حمل غير آمن، وليس بالضرورة فقط العنف الذي تعانيه من زوجها، يمكنها الإبلاغ عن حالتها عبر الإنترنت بشكل مجاني. " |
| (MW-06, Sana’a Gov, rural, aged 30-39) | "I have never heard of these services before. There are no safe spaces for women here, maybe because I live in a small village." | "لم اسمع من قبل بهذه الخدمات. لا يوجد مساحات آمنة لنساء هنا ربما لأني أعيش في قرية صغيرة….." |
| . (MW-02, Amintasmiah, urban, aged 30-39) | Interviewer: “Have you reported or referred a battered woman to the hotline?  Respondent: No one would call; women are afraid to report.  Interviewer: How are they helped by the hotline?  Respondent: It only provides counselling.  Interviewer: Are they referred to hospitals?  Respondent: I don't know exactly what they do, but this hotline provides services and counselling, as I have heard... I don't know if anyone has benefited or not, because most of the cases disappear after the first time we see them.” | "المُحاور: هل قدمتَ بلاغًا أو أحالتَ امرأة معنّفة إلى الخط الساخن؟  المُجيب: لا أحد سيتصل؛ النساء يخافن من التبليغ.  المُحاور: كيف يتم مساعدتهن عن طريق الخط الساخن؟  “المُجيب: يُقدم فقط الإرشاد النفسي  المُحاور: هل يتم إحالتهن إلى المستشفيات؟  المُجيب: لا أعرف بالضبط ماذا يفعلون، ولكن هذا الخط الساخن يُقدم خدمات وإرشادًا، كما سمعت... لا أعرف ما إذا كان أي شخص استفاد أم لا، لأن معظم الحالات تختفي بعد المرة الأولى التي نراها فيها. " |
| **Barriers to accessing VAWG services** | | |
| MW-13, Taiz, urban, aged 40-49) | “Women are afraid to expose their bodies or claim their rights if they have experienced violence. I believe that solutions must be found to help women overcome their fears.” | "النساء يخافن من الذهاب وكشف جسدهن أو استرجاع حقوقهن إذا تعرضن للعنف. أؤمن بأنه يجب البحث عن حلول لكي تستطيع النساء التغلب على مخاوفهن."\|. |
|  | “In war-torn countries, addressing violence is considered less important than saving lives. This is the perspective of humanitarian organisations. As a result, support is primarily limited to emergency response.” (MW-01, Amanatasimah, urban, aged 40-49) | "في البلدان المنكوبة بالحروب، يُعتبر التصدي للعنف أقل أهمية مقارنة بإنقاذ الأرواح. هذه وجهة نظر المنظمات الإنسانية. ونتيجة لذلك، يقتصر الدعم بشكل أساسي على الاستجابة الطارئة." |
| (MW-07, Amanat Al Asimah, urban, aged 40-49). | “Look, an ordinary person cannot access these services, so organizations should promote and advertise these services. If we do statistics on the extent of women's knowledge of the services provided by the Yemeni Women's Union, the percentage will not exceed 10%.” | "الشخص العادي، البسيط، لا يستطيع الوصول إلى مثل هذه الخدمات، لذلك من الضروري على المنظمات الترويج والإعلان عن هذه الخدمات؛ إذا قمنا بإجراء إحصاءات حول مدى معرفة النساء بخدمات اتحاد نساء اليمن، فإن النسبة لن تتجاوز ١٠٪." |
| . (MW-02, Amanat al-Asimah, urban, age 25-34) | “Any course related to gender-based violence requires a complex process through the Ministry of Health to be implemented because society does not accept these things.” | "أي دورة تتعلق بالعنف القائم على النوع الاجتماعي تتطلب الكثير من الإجراءات في وزارة الصحة لتنفيذها لأن المجتمع لا يقبل هذه الأشياء." |
| (MW-07, Amanatasimah, urban, aged 40-49) | “If a woman experiences violence, she may not go to the hospital due to inhumane treatment, so women often choose silence over facing humiliation.” | "إذا تعرضت امرأة للعنف، قد لا تذهب إلى المستشفى بسبب المعاملة اللا إنسانية، لذا غالبًا ما تختار النساء الصمت على أن تتعرض للإهانة." |
| (MW-07, Amanatasimah, urban, aged 40-49). | “Look, an ordinary person cannot access these services, so organisations should promote and advertise these services. If we do statistics on the extent of women's knowledge of the services provided by the Yemeni Women's Union, the percentage will not exceed 10%.” | "الشخص العادي، البسيط، لا يستطيع الوصول إلى مثل هذه الخدمات، لذلك من الضروري على المنظمات الترويج والإعلان عن هذه الخدمات؛ إذا قمنا بإجراء إحصاءات حول مدى معرفة النساء بخدمات اتحاد نساء اليمن، فإن النسبة لن تتجاوز ١٠٪." |

MW=Midwife; VAWG=violence against women and girls
